# Reconstruction of the Posterior Lamella of the Lower Eyelid Using a Long L-Shaped Periosteal Flap: Technical Modification and Literature Review

**DOI:** 10.1055/a-2621-7781

**Published:** 2025-07-23

**Authors:** Hikaru Kono, Fumio Onishi, Yu Kagaya, Hiroto Obata

**Affiliations:** 1 Department of Plastic and Reconstructive and Aesthetic Surgery, Saitama Medical Center, Saitama Medical University, Saitama, Japan; 2 Department of Ophthalmology, Saitama Medical Center, Saitama Medical University, Saitama, Japan

**Keywords:** lower eyelid defect, eyelid tumors, periosteal flap, myocutaneous advancement flap, frontozygomatic suture

## Abstract

Free tarsal grafts, the palatal mucosa, and auricular cartilage are commonly used in the reconstruction of the posterior lamella of the eyelid. However, reports describing the sole use of periosteal flaps are limited. We described the cases of two female patients, aged 72 and 85 years, with sebaceous gland and basal cell carcinomas of the left lower eyelids, respectively, who underwent reconstruction with a long L-shaped periosteal flap. The periosteal flap, measuring approximately 6 × 25 mm, was harvested along the vertical axis over the lateral orbital rim, extending across the frontozygomatic suture with the pivot positioned posteriorly at Whitnall's tubercle. This technique enabled the reconstruction of the posterior lamella of the lower eyelid. At the 1-year follow-up, mild sagging of the reconstructed area was observed in the second case; however, no major complications occurred. Thus, the long L-shaped periosteal flap was useful for reconstructing the lateral lower eyelid.

## Introduction

The reconstruction of the posterior lamella of the eyelid commonly utilizes free tarsal grafts, the palatal mucosa, and auricular cartilage; however, reports on the use of periosteal flaps are limited.

We report two cases (cases 1 and 2) of posterior lamellar defects of the lateral lower eyelid reconstructed with a periosteal flap in an L-shaped configuration, extending cranially across the frontozygomatic (FZ) suture.

## Case

**Fig. 1:**
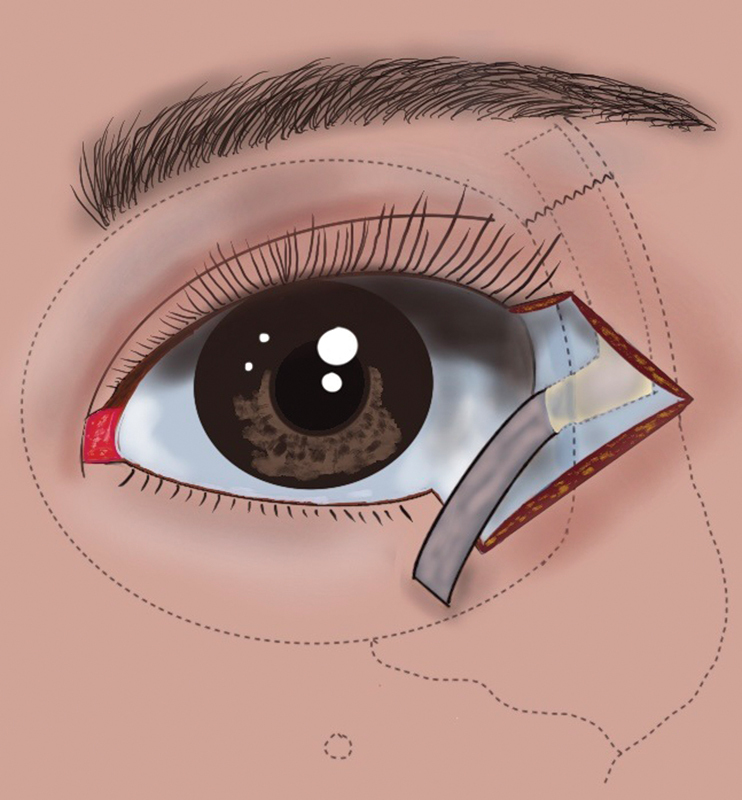
Design of the long L-shaped periosteal flap. The flap is designed on the lateral orbital rim and extends cranially across the frontozygomatic suture (dotted line). Note that the base of the flap can be extended posteriorly over Whitnall's tubercle in an L-shape configuration.

The surgical procedures were common to cases 1 and 2. The tumor was resected with a 3-mm surgical margin, which was confirmed to be negative histopathologically. The lateral orbital rim superior to the FZ suture was exposed beneath the orbicularis muscle, and a periosteal flap was raised from the superolateral orbital rim. Subperiosteal dissection proceeded in a superior-to-inferior direction, pivoting just above Whitnall's tubercle ( Fig. 1 ). The periosteal flap, measuring up to 25 mm in length and 6 mm in width, was trimmed and sutured to the remaining tarsus. We further dissected the base of the flap in a posterior direction over Whitnall's tubercle for intimate contact between the reconstructed eyelid and ocular surface. The residual conjunctiva was sutured to the inferior edge of the transposed periosteal flap, which was covered with the skin flap advanced to the upper edge of the periosteal flap.

In cases with anatomical variations, such as age-related periosteal thinning, additional techniques are needed. If the periosteal flap is first incised along the medial edge, it may retract laterally, making the lateral incision challenging. Therefore, beginning with the incision of the lateral edge and proceeding to the medial and superior edges facilitates smoother elevation of the periosteal flap. Additionally, thinner flaps may provide inadequate support to the lower lid. In such cases, a longer flap allows excess periosteum to reinforce structural stability.

**Fig. 2:**
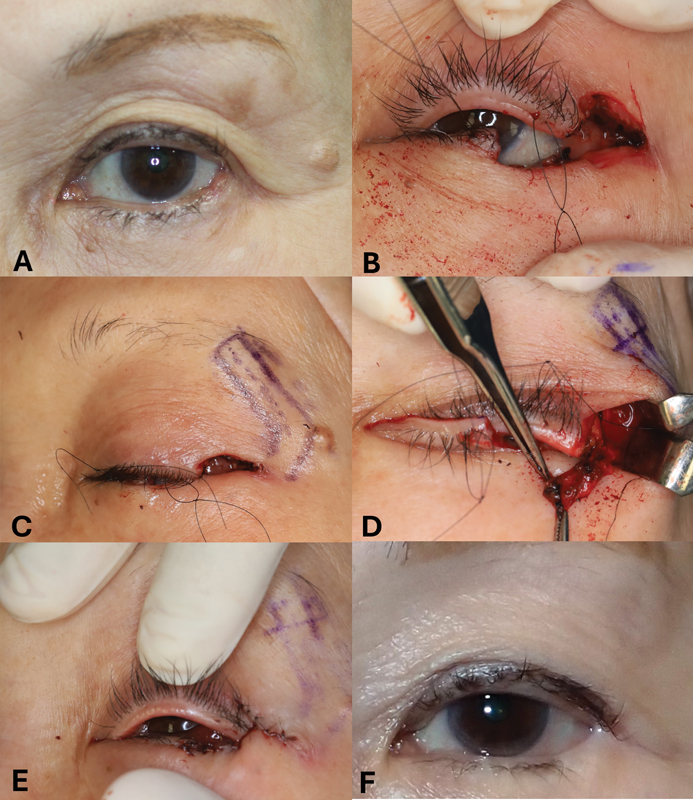
Intraoperative findings of case 1. **(A)** A preoperative photograph of a patient with a sebaceous gland carcinoma on the left lower eyelid. The tumor involved the left lateral canthal area. **(B)** The tissue defect after tumor resection involves the lateral upper and lower eyelids. **(C)** A long L-shaped periosteal flap was designed on the skin. **(D)** The elevated periosteal flap. **(E)** The defect of the anterior lamella was reconstructed with the remaining eyelid skin flap advancement over the periosteal flap. **(F)** A photograph obtained 1 year after surgery.

Case 1 included a 72-year-old female patient with a sebaceous gland carcinoma of the left lower eyelid, resulting in a defect width of 10 mm, accounting for 38% of the eyelid width ( Fig. 2A, B ). The periosteal flap was elevated ( Fig. 2C, D ), and the anterior lamella was reconstructed by suturing the remaining eyelid skin directly to the periosteal flap ( Fig. 2E ).

**Fig. 3:**
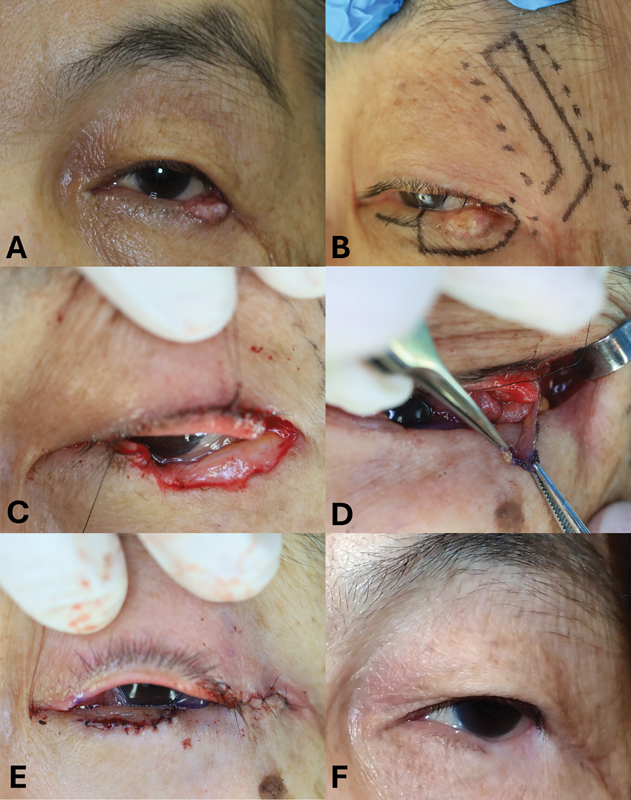
Intraoperative findings of case 2. **(A)** A preoperative photograph of a patient with a basal cell carcinoma on the left lower eyelid. The tumor involved the left lateral canthal area. **(B)** A long L-shaped periosteal flap was designed on the skin. **(C)** The defect was after wide resection of the tumor, which involved the lateral upper and lower eyelids. **(D)** The elevated periosteal flap. **(E)** The anterior lamella was reconstructed with a myocutaneous advancement flap. **(F)** A photograph obtained 1 year after surgery. Note that a mild sagging of the reconstructed area was observed.

Case 2 included an 85-year-old female patient with basal cell carcinoma of the left lower eyelid with a defect width of 44% ( Fig. 3A, B ). Similarly, the periosteal flap was elevated ( Fig. 3C, D ). To reconstruct the anterior lamella with vertical skin loss of 8 mm, an orbicularis oculi myocutaneous flap was raised from an additional subciliary incision down to the inferior orbital rim. The myocutaneous advancement flap was sutured directly to the transposed periosteal flap and the lateral orbital rim to reconstruct the lid margin while preventing ectropion ( Fig. 3E ).

At the 1-year follow-up, both patients exhibited a cosmetically acceptable postoperative appearance ( Figs. 2F and 3F ); however, the patient in case 2 showed mild lower eyelid sagging. Neither patient developed complications, such as ectropion, blunting of the lateral canthal angle, suture line notching, or wound dehiscence. Because the reconstruction was performed using a periosteal flap, thinning of the reconstructed area was observed in both cases. According to the seventh edition of the American Joint Commission on Cancer tumor (T), lymph node (N), and metastasis (M) classification, the tumors in cases 1 and 2 were Stage 1b (T2a, N0, M0).

## Discussion

We developed a modified technique for reconstructing the posterior lamella of the lower eyelid using a periosteal flap with an L-shaped extension. Periosteal flaps have been previously used for reconstructing the lower eyelid. 1 Weinstein et al. recommended a minimum flap width of 1 cm and sufficient length to reach the residual tarsus. 1 We modified previously reported procedures to harvest a longer periosteal flap to reach the residual tarsus. The periosteal flap can be harvested from the lateral or inferior orbital rims. However, the periosteal flap from the lateral orbital rim provides better lower eyelid support, as it suspends the reconstructed lower eyelid toward the lateral canthal ligament while reflecting inferomedially. Furthermore, as a result of the posterior extension, the flap could pivot on a more posterior point to provide a reconstructed eyelid with tight intimacy to the ocular surface, which also provides the reconstructed eyelid with enough length and support.

Reconstruction of full-thickness lower eyelid defects typically involves primary closure for defects up to 30% of the eyelid, and lateral cantholysis or a Tenzel flap for defects less than 50%. 2 3 However, when using a Tenzel flap, residual tarsus must be present on the lateral side of the defect. In the absence of residual tarsus, reinforcement with a periosteal flap is required. 4 For defects exceeding 50%, posterior lamella reconstruction with substitute tissues such as a Hughes flap, palatal mucosa, auricular cartilage, free tarsal graft, or periosteal flaps becomes necessary. 2 5

Previous reports on periosteal flaps for posterior lamella reconstruction have predominantly utilized flaps harvested transversely across the lateral orbital rim. 1 4 6 7 8 9 10 11 In cases where the periosteal flap length is insufficient, some reports have described extending the flap to the temporalis fascia, while others have suggested combining with a free tarsal graft. 1 6 However, extending the flap to the temporalis fascia risks injuring the temporal branch of the facial nerve due to its anatomical course. Using additional free tarsal grafts necessitates harvesting tissue from an additional donor site. Ongkasuwan harvested a periosteal flap by extending it superiorly from the lateral orbital rim, closely resembling our approach. 12 Their study reported the feasibility of harvesting a periosteal flap measuring 6 to 8 mm in width and 15 to 20 mm in length. In our method, we modified the periosteal flap into an L-shape configuration at its base and extended the flap tip beyond the FZ suture upward, enabling the harvest of a 25-mm-long flap.

Reconstruction of the posterior lamella using a periosteal flap is relatively safe and yields good cosmetic outcomes. Weinstein et al. reported only minor complications, including ectropion, lower lateral retraction, blunting of the lateral canthal angle, eyelid notching and dehiscence, and symblepharon formation in 11 patients who underwent upper and lower eyelid reconstruction using the periosteal flap. 1 Blumenthal et al. reported mild to moderate complications in 4 of 10 patients, including foreign body sensation, slight scleral show, trichiatic lower eyelashes, canthal webbing, lagophthalmos, and ectropion. However, no serious complication was observed. 7 Álvaro Toribio reported that only one case developed a granuloma out of nine comparable cases. 8 Leone and Perry and Allen did not observe any complications in patients who underwent lateral lower eyelid reconstructions with periosteal flaps. 5 9 Similarly, Balchev and Murgova and Ongkasuwan 12 reported no long-term complications or the need for secondary surgical interventions in their patients. 6 10 The patient in case 2 exhibited sagging of the reconstructed lower eyelid, likely due to a larger vertical dimension of the defect than that in case 1. This suggests that using a lateral skin flap may lower the risk of sagging in cases with larger vertical defects. In addition, the second patient was older than the first, suggesting the possibility of age-related horizontal laxity of the lower eyelid. The periosteal flap appeared thinner in the second case, indicating that the strength of the periosteal flap might have decreased owing to aging.

Harvesting the periosteal flap in an L-shape configuration allows for obtaining a longer flap compared with conventional methods, thereby expanding the range of posterior lamella reconstruction. Additionally, sufficient flap length offers adjustability to the required size and excess periosteum to reinforce structural support. While our method demonstrated reconstructing defects up to 44% of the lid width, the actual flap length theoretically allows for the reconstruction of defects exceeding 50%. However, as long periosteal flaps may provide less structural support than palatal mucosa or auricular cartilage, reconstructing the anterior lamella using a myocutaneous advancement flap from the lower eyelid may not be ideal for extensive defects. In such cases, other techniques such as the cheek rotation flap should be considered for anterior lamella reconstruction. 13

**Fig. 4:**
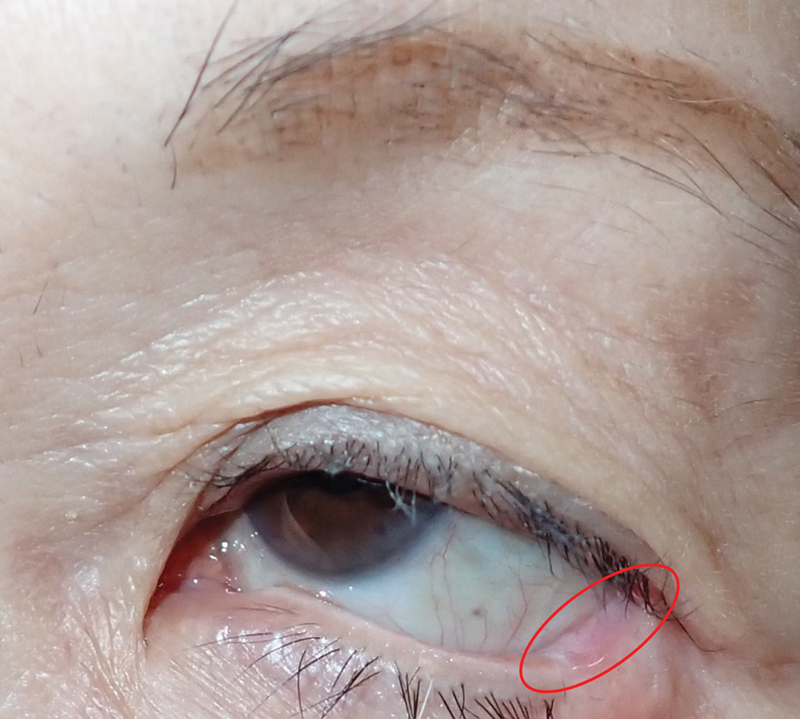
Epithelialization on the conjunctival surface of the periosteal flap. The conjunctival surface of the periosteal flap shows excellent epithelialization (red circle).

There are some limitations to our case series. First, the number of presented cases is small. We need further experience with the L-shaped periosteal flap technique to confirm the long-term efficacy for lower eyelid reconstruction. Second, we did not have direct evidence of the blood supply of the long periosteal flap; however, based on the successful epithelialization on the conjunctival surface of the periosteal flap in our series, the flap is indicated to have had enough vascular supply to encourage conjunctival healing ( Fig. 4 ). To secure flap survival, the periosteal flap should be covered with a vascular-rich skin flap, such as an orbicularis oculi myocutaneous flap. In addition, a microenvironment moisturized with lacrimal fluid might promote wound healing. Despite these limitations, we showed that the periosteal flap completely survived without major complications.

In conclusion, the L-shaped long periosteal flap may be considered a useful reconstructive option for the posterior lamella of the lower eyelid.
